# Successful rescue of a rare case of refractory shock due to multidrug toxicity: a case report

**DOI:** 10.3389/fphar.2025.1669831

**Published:** 2025-09-02

**Authors:** Yuhua Xie, Yujie Zhou, Yunxing Cao

**Affiliations:** ^1^ Department of Critical Care Medicine, The Second Affiliated Hospital of Chongqing Medical University, Chongqing, China; ^2^ Department of Critical Care Medicine, Chongqing General Hospital, Chongqing, China

**Keywords:** reserpine, metformin-associated lactic acidosis, intractable shock, hypoglycemia, multiple organ dysfunction, multidisciplinary diagnosis and treatment

## Abstract

**Introduction:**

This article reports the successful treatment experience of refractory shock, acidosis, hypoglycemia, and multi-organ failure after overdose of reserpine, enalapril, and metformin, and aims to provide a reference for the rescue of patients with such poisoning.

**Methods:**

A 58-year-old woman with sudden disturbance of consciousness, intractable shock, severe metabolic acidosis, hypoglycemia, and multiple organ dysfunction was urgently transferred to the ICU. The blood pressure could not be maintained after massive rehydration and a large dose of vasoactive drugs. The treatment plan was adjusted quickly after the diagnosis and treatment of medicine, nephrology, cardiology, respiratory, and other disciplines, such as high-dose vasoactive drugs, pressors, continuous blood purification, plasma exchange and hemoperfusion. RESULTS: The dose of vasoactive drugs began to decrease after entering the ICU at 36 h, and the acidosis gradually improved after 46 h. After 72 h, the patient was conscious and was transferred to the general ward on the 9th day.

**Conclusion:**

The treatment of mixed drug poisoning involving reserpine, metformin, and enalapril is very challenging. When resuscitating patients with refractory shock caused by such poisoning, early supportive therapies should be started, including gastric lavage, cathartic use, acid correction, improved ventilation, blood purification, and hemodialysis when needed. Additionally, vasoactive drugs may be given without an upper dose limit, as long as side effects are carefully monitored.

## 1 Introduction

Reserpine is an adrenergic nerve blocker that produces antihypertensive effects by depleting adrenaline from peripheral sympathetic nerve endings, as well as slowing the heart rate and depressing the central nervous system, with overdosage leading to hypotension, coma, and respiratory depression (Shamon and Perez, 2016). It is a gradually forgotten antihypertensive drug, and reserpine poisoning is now extremely rare.

Metformin is a first-line medication for the treatment of type 2 diabetes and typically does not cause hypoglycemia. Metformin-associated lactic acidosis (MALA) is a rare but severe metabolic complication associated with metformin use. The annual incidence of MALA in diabetic patients is less than 0.01–0.09 per 1,000 individuals, with a mortality rate as high as 50% (Calello et al., 2015; Li et al., 2021). This article reports a case of refractory shock caused by overdose of the above drugs. Through a summary of the treatment of this patient, we hope to provide new insights for the treatment of similar patients.

## 2 Case report

### 2.1 Description

The patient is a 58-year-old woman admitted to the orthopedic department for lower back pain. On 28 June 2022, her family discovered she was unresponsive and unable to communicate, with no signs of urinary or fecal incontinence. Dry, pinkish vomit with white powdery residue was observed at the bedside. Physical examination: Temperature 36 °C, heart rate 81 beats per minute, respiratory rate 23 breaths per minute, pulse oxygen saturation 94% (on oxygen at 3 L/min). Blood pressure measured multiple times was consistently 50–60/20–30 mmHg. Bilateral pupil diameter 4 mm, with delayed light reflex. The neck was soft with no resistance. Bilateral lung sounds were clear with no rales heard. Heart rhythm was regular with no murmurs heard. The abdomen is soft, and the liver and spleen have not been palpated. Both lower limbs showed no edema, and there was no movement in the limbs upon provocation. The patient was immediately administered dopamine intravenous infusion for blood pressure elevation and fluid infusion therapy, and was urgently transferred to the ICU.

### 2.2 Physical examination and lab results upon admission to the ICU

Upon admission to the ICU, the patient was in a comatose state, with unequal pupil sizes (left 3 mm, right 4 mm), absent light reflexes, deep and rapid breathing, and an oxygen saturation of approximately 94% on nasal cannula oxygen therapy. Blood pressure was 57/38 mmHg. Arterial blood gas analysis: pH 7.17, PO_2_ 106 mmHg, PCO_2_ 39 mmHg, cHCO_3_^–^ 14.2 mmol/L, BE -13.6 mmol/L, lactate 9.8 mmol/L, FiO_2_ 41%. Complete blood count: white blood cell count 16.04 *10^9^/L, neutrophil percentage 83.9%, hemoglobin count 117 g/L, platelet count 183*10^9^/L, high-sensitivity C-reactive protein <0.5 mg/L; renal function: creatinine 145.1 μmol/L, blood urea nitrogen 5.05 mmol/L, estimated glomerular filtration rate 34.0 mL/min; blood glucose 2.5 mmol/L; Negative for ketones, liver function is normal.

### 2.3 Intervention

Upon admission to the ICU, we immediately initiated endotracheal intubation with mechanical ventilation, aggressive fluid resuscitation, and continuous infusion of norepinephrine (NE) at 2–3 μg/(kg·min), dopamine (DA) at 20–30 μg/(kg·min), and dobutamine at 5–10 μg/(kg·min), epinephrine (E) 1.0–1.5 μg/(kg·min), metaraminol 0.1–0.2 mg/min, and posterior pituitary hormone at 0.1–0.2 IU/min to maintain blood pressure. The patient’s blood pressure was 85–96/40–50 mmHg, with persistently elevated lactate levels (peak lactate 31 mmol/L) and severe metabolic acidosis (pH 7.0–7.1) (Table 1), along with recurrent hypoglycemia (lowest 2.2 mmol/L). Pulse indicator continuous cadiac output (PICCO) showed a cardiac index (CI) of 4.28–4.29 L/min/m^2^, global end diastolic volume (GEDV) of 664.8–817.9 mL/m^2^, and extravascular lung water (EVLW) of 9.8–12.1 mL/kg, pulmonary vascular permeability index (PVPI) of 2.1–2.4, and systemic vascular resistance (SVR) of 766–1,027 dyn·s·cm^^(−5)^·m^2^, suggesting high output and low resistance. The patient presented with altered consciousness and shock of unknown etiology, accompanied by severe metabolic acidosis, recurrent hypoglycemia, hyperlactatemia, and acute renal failure. PICCO indicated distributive shock. Given the patient’s recent history of depressed mood and the presence of packaging boxes for reserpine, enalapril, and metformin, along with dried pinkish-white powdery vomitus found at the bedside, the likelihood of mixed drug poisoning was considered extremely high. Following a multidisciplinary treatment (MDT) involving the pharmacy department, nephrology, cardiology, and respiratory medicine, the treatment plan was rapidly adjusted, with the following measures implemented: (1) gastric lavage and cathartic administration; (2) fluid resuscitation (including crystalloid solutions, human albumin, and plasma); with daily fluid administration adjusted based on PICCO results and bedside critical care ultrasound-measured inferior vena cava distension rate; (3) Administration of high-dose vasoactive medications to maintain mean arterial pressure around 65 mmHg, combined with hydrocortisone; (4) Continuous renal replacement therapy (CRRT) using continuous veno-venous hemofiltration mode, combined with daily plasma exchange and two sessions of hemoperfusion. (5) Implement brain protection therapy; (6) Maintain electrolyte balance. During the patient’s ICU hospitalization, the patient developed critical conditions such as hypothermia, disseminated intravascular coagulation (DIC), respiratory failure, pulmonary edema, paralytic ileus, gastrointestinal bleeding, airway bleeding, myocardial injury, capillary leak syndrome, and abdominal compartment syndrome, with pupil diameter even reaching 7 mm. Considering the patient had taken a large dose of reserpine, leading to depletion of catecholamine hormones, she continued to receive large doses of norepinephrine and epinephrine.

**TABLE 1:** Selected lab values, arterial blood gas (ABG) parameters, vasopressor infusion rates, and blood pressure (BP) measurements.

Parameter	Adm	T = 0 h	T = 4 h	T = 12 h	T = 24 h	T = 48	T = 72 h	Normal	Unit
PH	-	7.17	7.14	7.17	7.14	7.29	7.35	7.35–7.45	-
Lac	3.41	11.24	24.01	-	24.28	30.6	9.4	1.32–3.96	mmol/L
BE	-	−13.6	−17.8	−16.9	−17	−9.8	−6	0 ± 3	mmol/L
HCO3-	21.9	14.2	9.9	10.2	10.6	15.9	18.8	21–26	mmol/L
ALT	8	12	43	-	264	91	69	7–40	U/L
AST	13	23	61	-	454	181	141	13–30	U/L
BUN	4.90	5.05	4.46	-	4.46	8.04	9.22	2.6–7.5	mmol/L
Cr	61.9	145.1	43.2	-	87	136.8	155.9	41–73	μmol/L
GFR	95.8	34.3	44.1	-	63.6	36.8	31.4	80–300	ml/min
NE	-	-	1.5	2	2.5	1.2	0.6	-	μg/kg*min
E	-	-	0.03	0.1	1.5	0.27	-	-	μg/kg*min
DA	-	-	5	20	10	8	-	-	μg/kg*min
DBT	-	-	-	-	-	3	-	-	μg/kg*min
PTT	-	-	3	10	19	8	-	-	iu/h
Aramine	-	-	-	-	12	6	-	-	mg/h
HC	-	-	-	-	16	-	-	-	mg/h
Bp	-	68/31	93/50	85/40	94/54	142/77	132/79	-	mmHg

Adm, admission to hospital; Lac, lactate; BE, base excess; ALT, alanine aminotransferase; AST, aspartate aminotransferase; BUN, blood urea nitrogen; Cr, creatinine; GFR, glomerular filtration rate; NE, norepinephrine; E, epinephrine; DA, dopamine; DBT, dobutamine; PTT, pituitrin; HC, hydrocortisone; Bp, blood pressure.

### 2.4 Prognosis

Vascular active drugs were gradually tapered off 36 h after admission to the ICU and discontinued on the 7th day. Acidosis improved 46 h after admission, with a pH value of 7.25, and was corrected 56 h later, with a normal pH value. Lactate levels began to decrease 48 h after admission, reaching 9.4 mmol/L at 72 h, and returned to normal on July 4. The patient’s consciousness gradually improved 48 h after admission, and the patient was fully conscious 72 h later. And the pupil diameter returned to 3 mm. The endotracheal tube was removed on the 7th day after admission to the ICU, and the patient was transferred to a general ward on the 9th day. After the patient regained consciousness, she was questioned and reported that she had taken one bottle of reserpine tablets (approximately 100 tablets/25 mg), one box of metformin extended-release tablets (approximately 30 tablets/15 g), and one box of enalapril maleate tablets (approximately 16 tablets/80 mg). At discharge, the patient was fully conscious, with normal liver function and urine output.

## 3 Discussion

A review of domestic and international literature revealed no reports of refractory hypotension, hypoglycemia, and multiple organ failure caused by the simultaneous use of the three drugs in this case. The patient was concurrently taking large doses of reserpine, enalapril, and metformin, which interacted in complex ways, leading to refractory shock and multi-organ failure within a short period. The case had the following characteristics: (1) Refractory shock, with blood pressure barely maintained at the lower limit of normal levels despite aggressive fluid resuscitation and the use of high doses of vasoactive drugs; and the doses of norepinephrine and dopamine used exceeded the conventional range; (2) Persistent severe metabolic acidosis, with a pH value of only 7.0–7.1 despite early use of CRRT and peripheral infusion of sodium bicarbonate, and persistently elevated lactate levels, with the highest lactate level reaching 31 mmol/L; (3) Recurrent hypoglycemia (lowest 2.2 mmol/L); (4) Drug-induced encephalopathy combined with ischemic-hypoxic encephalopathy, with persistent coma for 3 days, and pupil dilation to 7 mm in diameter, which persisted until 72 h after admission to the ICU before returning to 3 mm; (5) acute renal failure; (6) hypothermia; (7) disseminated intravascular coagulation (DIC), gastrointestinal bleeding, and airway bleeding; (8) respiratory failure, pulmonary edema; (9) paralytic ileus; (10) capillary leak syndrome; (11) abdominal compartment syndrome; (12) toxic myocardial injury.

Metformin is rapidly metabolized by the kidneys. MALA primarily occurs in patients with significant renal dysfunction. In such patients, early manifestations are primarily gastrointestinal reactions, such as nausea, vomiting, abdominal pain, diarrhea, and loss of appetite (Angeletti et al., 2023). In this case, the patient developed significant impaired consciousness and severely elevated lactate levels after taking 15 g, which was considered to be related to drug interactions. Persistent shock induced by reserpine overdose leading to prerenal AKI, combined with the nephrotoxic effects of captopril, both become predisposing comorbidities for lactic acidosis. Deterioration of renal function leads to drug accumulation, promoting the progression of lactic acidosis. Lactic acidosis further causes a decrease in vascular tone, resulting in a vicious cycle of shock-acidosis-shock. Treatment for MALA patients currently focuses on removing gastrointestinal toxins, correcting acidosis with sodium bicarbonate, improving ventilation to ensure oxygen supply, and intermittent hemodialysis to remove toxins (Manouchehri et al., 2023). In recent years, CRRT has been applied to MALA, but the optimal timing for initiating CRRT remains unclear. Some scholars suggest initiating CRRT treatment when lactate levels exceed 20 mmol/L, pH is below 7, shock is present, standard supportive measures fail, and consciousness deteriorates (Calello et al., 2015).

The mechanism by which metformin lowers blood glucose levels includes reducing hepatic gluconeogenesis, decreasing glucose absorption in the small intestine, and increasing peripheral tissue uptake and utilization of glucose. Since metformin does not stimulate insulin secretion, patients receiving metformin monotherapy typically do not experience hypoglycemia under normal circumstances. However, hypoglycemia may occur in patients who eat insufficiently, engage in excessive physical activity, or use metformin in combination with other antidiabetic medications. In this patient, hypoglycemia occurred despite not using other hypoglycemic agents, which may be related to excessive use of metformin, leading to increased anaerobic glycolytic glucose consumption. Recent literature has also reported cases of recurrent hypoglycemia after taking 30 g of metformin alone (Aldobeaban et al., 2018). Additionally, studies have shown that ACEI inhibitors can improve insulin sensitivity, increase insulin secretion, and improve insulin resistance. The patient was also taking a large dose of enalapril, which may have interacted with metformin to cause recurrent hypoglycemia (Zhang et al., 2007).

Through reviewing the literature and our experience in treating and rescuing this patient, we have summarized the following points: (1) Early initiation of active multidisciplinary supportive therapy: For drug poisoning, especially with drugs like reserpine that lack specific antidotes, the treatment focus is on promptly removing the toxin and providing symptomatic supportive care. Gastric lavage and cathartic agents should be used to rapidly expel any residual drug not yet absorbed from the gastrointestinal tract, thereby reducing reabsorption by the intestinal mucosa and lowering the toxic dose (Feng et al., 2010; Ma and Li, 2019). Early initiation of continuous renal replacement therapy (CRRT) combined with hemoperfusion and plasma exchange is essential to correct internal environmental disorders, remove drugs and inflammatory mediators from the body, alleviate pulmonary edema, and strive for early restoration of renal function. Metformin and enalapril can both be removed via hemodialysis. Reserpine cannot be removed by dialysis, but it has a high plasma protein binding rate, so active hemoperfusion and plasma exchange can be performed to accelerate toxin clearance; additionally, effective mechanical ventilation support is also required. (2) Safe use of high-dose vasoactive drugs under close monitoring: The patient has refractory shock, and reserpine leads to the exhaustion of catecholamine hormones in the body, so a large amount of NE and E needs to be supplemented. Some institutions set maximum doses for pressor drugs due to the patient’s severe condition, high risk of death, and to control drug side effects. This patient received extra-high doses of NE and DA without experiencing side effects such as peripheral ischemia, coronary ischemia, or arrhythmia. This phenomenon demonstrates that the dosage of vasoactive drugs can be adjusted without setting a maximum dose, provided that side effects are closely monitored. (3) Lactate is not a decisive indicator for assessing the prognosis of this type of patient: Lactate levels are an important indicator for assessing prognosis and are closely related to mortality rates; However, they must be interpreted on an individual basis. In this patient, lactate levels reached 31 mmol/L, which was attributed not only to hypoperfusion caused by refractory shock but also to the effects of MALA. Therefore, when treating patients with MALA, one should not prematurely abandon resuscitation efforts solely based on elevated lactate levels. (4) If circulation cannot be maintained with conventional resuscitation measures and the condition continues to deteriorate, extracorporeal membrane oxygenation (ECMO) therapy may be required. Previous studies have reported that ECMO-provided organ support can rapidly improve physiological dysfunction and increase the success rate of resuscitation in cases of life-threatening drug-induced toxic shock (Weiner et al., 2020). For other patients with refractory cardiogenic shock and acute respiratory distress syndrome caused by drug poisoning, ECMO is an excellent life-saving measure. (5) Metformin therapy mandates strict renal surveillance. In patients with renal impairment, both initiation and dose selection demand caution, as sub-maximal doses can accumulate and raise MALA risk. Hence, all diabetic users should have eGFR rechecked annually (Silverii, 2024).

## 4 Conclusion

In summary, although the incidence of refractory shock, hypoglycemia, and MALA caused by mixed drug poisoning from reserpine, enalapril, and metformin is low, the mortality rate is high. The complex interactions and synergistic effects of multiple drugs can rapidly lead to multi-organ failure, and the complex hemodynamic changes increase the difficulty of treatment. When poisoning is detected, early measures such as gastric lavage, catharsis, hemodialysis, vasoactive drugs to maintain blood pressure, and other symptomatic supportive treatments should be used. Multidisciplinary care plays a significant role in the treatment process. For cases with refractory cardiogenic shock and refractory acute respiratory distress syndrome, ECMO support may be considered.

## Data Availability

The raw data supporting the conclusions of this article will be made available by the authors, without undue reservation.
